# Body Mass Index and Pregnancy Outcome after Assisted Reproduction Treatment

**DOI:** 10.1155/2014/257974

**Published:** 2014-03-05

**Authors:** Khaled Kasim, Ahmed Roshdy

**Affiliations:** ^1^Department of Public Health and Community Medicine, Faculty of Medicine, Al-Azhar University, Nasr City, Cairo, Egypt; ^2^Assisted Reproductive Technology (ART) Unit, International Islamic Centre for Population Studies and Research (IICPSR), Al-Azhar University, Cairo, Egypt

## Abstract

The present study aimed to evaluate the impact of body mass index (BMI) on pregnancy outcome after intracytoplasmic sperm injection (ICSI). The study analyzed pregnancy outcome of 349 women who underwent ICSI by their BMI: <25, 25–<30, and ≥30 kg/m^2^. The associations were generated by applying logistic regression models. A significant reduction in positive pregnancy outcome was observed among overweight and obese women (odds ratio (OR) = 0.50; 95% confidence interval (CI) = 0.25–0.99 for overweight women and OR = 0.45; 95% CI = 0.20–0.89 for obese women). These estimates show that the pregnancy rates are reduced with increasing BMI. The effect of obesity on pregnancy outcome was absent when three and more embryos were transferred. Our study contributes to the reports linking overweight and obesity with decreased positive pregnancy outcome after ICSI and suggests women's age, infertility type, and number of embryos transferred to modify this reducing effect.

## 1. Introduction

Body mass index (BMI) has an adverse effect on reproduction [1]. Overweight women have a higher incidence of menstrual dysfunction and anovulation, possibly because of altered secretion of gonadotropin releasing hormone, sex hormone binding globulin, ovarian and adrenal androgen, and luteinising hormone and also because of altered insulin resistance [2]. A body mass index that was either high or low was associated with reduced probability of achieving pregnancy in women receiving assisted reproduction treatment. Mechanisms through which body mass affects reproduction that have been cited include menstrual disturbance and anovulation [3] but these problems can be overcome through assisted reproduction treatment. There is no evidence that body mass affects the quality of the embryo and therefore the pregnancy rate. Other mechanisms may be proposed such as altered receptivity of the uterus after transfer of embryos, possibly because of disturbed endometrial function [2].

The prevalence of obesity in infertile women is high, and there is growing evidence that BMI is associated with pregnancy outcome after ART. Several recent and previous studies have linked overweight and obesity to low pregnancy rate [4–8] and spontaneous abortion [9] in ART programs. Obesity is also potentially modifiable, possibly amenable to low cost, noninvasive self-management by patients. These studies, however, did not examine the interaction (effect modification) between BMI and other related factors on the probability of pregnancy outcome in these women. According to published studies [10, 11], patient's age, infertility type, and number of transferred embryos appeared to be the most important factors affecting pregnancy outcome after ICSI. The current study aimed to examine the association of pregnancy outcome with BMI and to assess the possible effect modification of women's age, infertility type, and number of transferred embryos on the probability of positive pregnancy outcome associated with body mass index in infertile women receiving assisted reproduction treatment.

## 2. Methods

A retrospective study was designed to examine the association of pregnancy outcome with BMI and to examine the possible effect modification of women's age, infertility type, and number of transferred embryos on the observed association. The study extracted data from the files of 349 infertile women who underwent assisted fertilization at the ART Unit, the International Islamic Center for Population Researches and Studies (IICPRS), Al-Azhar University, during 2011. Three hundred forty-nine files were selected randomly from the ART medical data archives according to the estimated sample size. Because of missing outcome data, only one file was excluded from study analyses. The sample size is calculated according to the estimated average pregnancy rate in the studied center during the previous years (25%) and to an assumed precision of 0.04 with confidence interval of 95% and probability value of 0.05. According to pregnancy outcome, a nested case-control approach is designed where women with positive pregnancy outcome were considered as cases and those with negative pregnancy outcome as controls. The study design was approved by medical ethical committee at ART Unit. The collected data were managed and analyzed confidentially and anonymously.

The main outcome variable was the pregnancy outcome. Urine and/or serum Human Chorionic Gonadotrophin (HCG) measurement was routinely obtained on days 14–17 after embryo transfer, and positive pregnancy is confirmed following HCG rise. The main studied variables were categorized for the clarity of data analysis and presentation of results as follows: age is classified into three categories according to tertile distribution of the studied women: <27 years, 27–33 years, and >33 years; and three BMI subgroups were formed: <25 kg/m^2^ (normal), 25–>30 kg/m^2^ (overweight), and ≥30 kg/m^2^(obese) [12]. The number of transferred embryos was classified into three categories (one embryo transfer, two embryos transfer, and three and more embryos transfer).

The collected data were analysed by using the statistical analysis system (SAS) software package [13]. Chi-square and *t*-tests were used as appropriate to compare the studied women by BMI in relation to their characteristics. Logistic regression models were used to estimate odds ratios and their 95% confidence intervals for the association of positive pregnancy outcome and BMI. To explore the possible effect modification of patient's age, type of infertility, and number of embryo transfer on the probability of positive pregnancy outcome associated with BMI, we incorporated the main effect variables and their cross-product terms into logistic regression models for testing of interaction based on a multiplicative model. *P* values of Wald test <0.05 were considered as evidence of statistical interaction.

## 3. Results

Of the studied 349 women, 52% were overweight and 32% were obese. The positive pregnancy rate among the whole studied women was 21% (75 of 349). The positive pregnancy rate is varied by BMI where it was 33%, 22%, and 18% in normal, overweight, and obese women, respectively.  Table 1 presents the women characteristics by BMI strata. There were significant differences among BMI strata regarding age, infertility duration, cycle length, and luteinising hormone (LH) level, where the highest mean age, infertility duration, cycle length, and the lowest mean LH level were among obese women. On the other hand, no significant differences among the three BMI strata were observed regarding other studied factors.

Table 2 shows odds ratios (ORs) and their 95% confidence intervals for the association of positive pregnancy outcome with BMI in the unadjusted and adjusted logistic model. The results of both models were nearly the same with no confounding effects of the controlled confounders being observed. The odds ratio of positive pregnancy is found to be negatively and significantly associated with overweight and obesity. Among overweight and obese women, the probability of positive pregnancy is reduced by 50% and 55%, respectively, in overweight and obese women (OR = 0.50; 95% CI = 0.25–0.99 among overweight women; OR = 0.45; 95% CI = 0.20–0.99 among obese women).

Table 3 shows the adjusted ORs of positive pregnancy outcome and their 95% CIs for the two-way interactions between BMI and patient's age, infertility type, and ET. Although no relation was detected between BMI and pregnancy outcome among women aged 27–33 years, a varying degree of reduction was observed for other age groups. The highest reduction was among overweight women aged >33 years (80%) and obese women aged <27 years (77%). Also, there has been a reduction in pregnancy outcome among overweight and obese women with primary and secondary infertility with the highest and significant reductions being for primary infertile women (OR = 0.27; 95% CI = 0.15–0.98 for overweight, and OR = 0.35; 95% CI = 0.12–0.99 for obese women). The reduction in pregnancy rate among overweight and obese women who had one or two ET was not seen among women who had more than two embryos transferred. In this group, the probability of positive pregnancy outcome was increased by 1.6-fold (OR = 1.60; 95% CI = 0.74–4.68).

## 4. Discussion

This study contributes to the previous reports of decreased probability of positive pregnancy outcome in overweight and obese women after infertility treatment [4, 6–8]. The endocrinological and/or biochemical milieu associated with obesity, operating through a functional state such as insulin resistance, can create a hostile intraovarian or intrauterine environment for the oocytes or embryos. Also, plausibility for a causal link between obesity and reproductive hormones can be found in studies that link modest weight loss and a reduction of central fat to improved insulin sensitivity, resulting in ovulation and pregnancy in overweight infertile women [14]. Moreover, obesity is associated with alterations in carbohydrate and fat metabolism central to the development of insulin resistance. A diet with a high-glycemic index has been associated with infertility, fetal loss, congenital anomalies, and prematurity, as well as macrosomia. Greater carbohydrate intake and dietary glycemic load have been associated with an increased risk of infertility due to anovulation [15].

The striking negative association of positive pregnancy outcome with overweight and obesity, observed in our study as in previous ones [3, 5, 16, 17], directed us to examine if the probability of positive pregnancy varies according to other known risk factors. The results suggest the reducing effect of overweight and obesity on the probability of positive pregnancy outcome to increase more in primary infertile women and among those aged <27 years and more than 33 years. However, this reducing effect disappeared among women aged 27–33 years and those have had two ET. The practice of multiple embryos transfer in ART has also been suggested to decrease early pregnancy loss among normal and overweight women [18, 19]. The mechanism by which age, infertility type, and number of ET can modify the probability of positive pregnancy associated with overweight and obesity is unclear. But these results may open a number of avenues for subsequent research to shed more light on such mechanisms. However, there is a still controversy about the dangerous effect of transferring multiple embryos. Multiple birth is related to the number of transferred embryos and is associated with adverse fetal and infant outcomes, and they also pose increased risks of maternal morbidity and mortality [20, 21]. Accordingly, weight reduction among obese women undergoing ART may be of value in reducing the number of transferred embryos and hence reducing the associated complications. However, a recent study [22] reported that short-term weight loss was unrelated to positive *β*-hCG, clinical pregnancy, or live birth rates, particularly among obese and overweight women.

To our knowledge, this study is the first to search for the possible effect modification of some women's characteristics as well as the number of embryo transfer on the reported negative association between BMI and positive pregnancy outcome. A potential concern in this study is the lack of data, due to the retrospective nature of the study. Accordingly, other factors were not available about women lifestyle factors (such as smoking, physical exercise, and dietary habit) to investigate their modification effect on the probability of pregnancy outcome associated with BMI.

In summary, this study confirms the reported reducing effect of overweight and obesity on positive pregnancy outcome in infertile women after assisted reproduction treatment. Our study also suggests a possible effect modification of patient's age, infertility type, and number of ET on such association. The latter observation deserves further investigation.

## Figures and Tables

**Table 1:** Distribution of the studied women by body mass index in relation to their characteristics.

Characteristics	Body mass index (kg/m^2^)			*P* value
	<25 (*n* = 51)	25–<30 (*n* = 184)	≥30 (*n* = 114)	
Age (years)	27.3 ± 4.5	29.8 ± 5.4	32.8 ± 5.7	0.001∗
Infertility duration (years)	5.4 ± 3.9	7.5 ± 4.7	8.5 ± 5.3	0.001∗
Cycle length (days)	27.6 ± 3.4	28.9 ± 8.1	31.5 ± 13.2	0.03∗
FSH (MIU/mL)	6.5 ± 2.5	6.2 ± 2.9	6.4 ± 2.6	0.78
LH^†^	6.7 ± 3.4	5.3 ± 2.7	5.1 ± 2.4	0.004∗
PRL∗∗	18.1 ± 16.6	15.5 ± 10.1	15.4 ± 10.9	0.45
E2 (pg/mL)	76.7 ± 59.9	74.2 ± 55.8	70.1 ± 51.6	0.82
Endometrial thickness	10.5 ± 2.2	10.5 ± 2.2	10.7 ± 2.1	0.68
HCG days	13.2 ± 2.4	13.7 ± 2.6	13.2 ± 2.2	0.10
Number of embryo transfer	2.8 ± 1.0	2.8 ± 0.9	2.8 ± 0.9	0.99
Infertility type				
Primary	46 (15%)	158 (53%)	94 (32%)	0.41
Secondary	5 (10%)	26 (50%)	20 (40%)	

*Significant.

^†^Luteinizing hormone.

∗∗Prolactin hormone.

**Table 2:** Odds ratios (OR) and their 95% confidence intervals (CI) for the association of BMI with positive pregnancy outcome.

Studied factor	Positive pregnancy women	Negative pregnancy women	OR	95% CI	*P*-trend
Body mass index (kg/m^2^)∗					0.04
<25	17	34	1.00	Ref.	
25–<30	37	147	0.50	0.25–0.99	
≥30	21	93	0.45	0.21–0.95	
Body mass index (kg/m^2^)^†^					
<25	17	34	1.00	Ref.	
25–<30	37	147	0.50	0.25–0.99	
≥30	21	93	0.45	0.20–0.99	

*Unadjusted model.

^†^Model adjusted by age groups, type of infertility, number of embryo transfer, HCG days endometrial thickness, and hormonal profiles.

**Table 3:** Positive pregnancy outcome associated with body mass index (kg/m^2^) by age groups, infertility type, and number of embryo transfer (ET).

Studied factor	Body mass index (BMI) kg/m^2^						*P*-interaction
	<25		25–<30		≥30	
	OR	95% CI	OR	95% CI	OR	95% CI
Age groups							
<27 years	1.00	—	0.30	0.10–0.85	0.23	0.05–1.00	0.46
27–33 years	1.00	—	1.02	0.50–1.95	0.95	0.30–2.45	
>33 years	1.00	—	0.20	0.10–0.98	0.30	0.15–1.30	

Type of infertility							
Primary	1.00	—	0.27	0.15–0.98	0.35	0.12–0.99	0.76
Secondary	1.00	—	0.45	0.05–2.85	0.50	0.10–3.65	

Number of ET∗							
One ET	1.00	—	0.20	0.05–1.90	—	—	0.33
Two ET	1.00	—	0.50	0.12–2.56	0.45	0.10–2.89	
Three and more ET	1.00	—	1.60	0.74–4.68	0.98	0.20–3.89	

*Number of embryo transfer.

## References

[1] Frisch RE (1989). Body weight and reproduction. *Science*.

[2] Wang JX, Davies M, Norman RJ (2000). Body mass and probability of pregnancy during assisted reproduction treatment: retrospective study. *British Medical Journal*.

[3] Lake L, Power C, Cole TJ (1997). Women’s reproductive health: the role of body mass index in early and adult life. *Internal Journal of Obstetric Related Metabolic Disorders*.

[4] Luke B, Brown MB, Stern JE, Missmer SA, Fujimoto VY, Leach R (2011). Female obesity adversely affects assisted reproductive technology (ART) pregnancy and live birth rates. *Human Reproduction*.

[5] Bellver J, Ayllón Y, Ferrando M (2010). Female obesity impairs in vitro fertilization outcome without affecting embryo quality. *Fertility and Sterility*.

[6] van Swieten ECAM, van der Leeuw-Harmsen L, Badings EA, van der Linden PJQ (2005). Obesity and clomiphene challenge test as predictors of outcome of in vitro fertilization and intracytoplasmic sperm injection. *Gynecologic and Obstetric Investigation*.

[7] Vural B, Sofuoglu K, Caliskan E (2005). Predictors of intracytoplasmic sperm injection (ICSI) outcome in couples with and without male factor infertility. *Clinical and Experimental Obstetrics and Gynecology*.

[8] Shen S, Khabani A, Klein N, Battaglia D (2003). Statistical analysis of factors affecting fertilization rates and clinical outcome associated with intracytoplasmic sperm injection. *Fertility and Sterility*.

[9] Hamilton-Fairley D, Kiddy D, Watson H, Paterson C, Franks S (1992). Association of moderate obesity with a poor pregnancy outcome in women with polycystic ovary syndrome treated with low dose gonadotrophin. *British Journal of Obstetrics and Gynaecology*.

[10] Broekmans FJ, Kwee J, Hendriks DJ, Mol BW, Lambalk CB (2006). A systematic review of tests predicting ovarian reserve and IVF outcome. *Human Reproduction Update*.

[11] Luke B, Brown MB, Stern JE, Grainger DA, Klein N, Cedars M (2010). Effect of embryo transfer number on singleton and twin implantation pregnancy outcomes after assisted reproductive technology. *Journal of Reproductive Medicine*.

[12] World Health Organization (WHO) (2000). Obesity: preventing and managing the global epidemic. *World Health Organization Technical Report Series*.

[13] SAS Institute (1999). *Proprietary Software Release 8. 2*.

[14] Huber-Buchholz M-M, Carey DGP, Norman RJ (1999). Restoration of reproductive potential by lifestyle modification in obese polycystic ovary syndrome: role of insulin sensitivity and luteinizing hormone. *Journal of Clinical Endocrinology and Metabolism*.

[15] Chavarro JE, Rich-Edwards JW, Rosner BA, Willett WC (2009). A prospective study of dietary carbohydrate quantity and quality in relation to risk of ovulatory infertility. *European Journal of Clinical Nutrition*.

[16] Bellver J, Ayllón Y, Ferrando M (2010). Female obesity impairs in vitro fertilization outcome without affecting embryo quality. *Fertility and Sterility*.

[17] Rittenberg V, Seshadri S, Sunkara SK, Sobaleva S, Oteng-Ntim E, El-Toukhy T (2011). Effect of body mass index on IVF treatment outcome: an updated systematic review and meta-analysis. *Reproductive BioMedicine*.

[18] Simón C, Landeras J, Zuzuarregui JL, Martín JC, Remohí J, Pellicer A (1999). Early pregnancy losses in in vitro fertilization and oocyte donation. *Fertility and Sterility*.

[19] Fedorcsák P, Storeng R, Dale PO, Tanbo T, Åbyholm T (2000). Obesity is a risk factor for early pregnancy loss after IVF or ICSI. *Acta Obstetricia et Gynecologica Scandinavica*.

[20] Reynolds MA, Schieve LA, Jeng G, Peterson HB, Wilcox LS (2001). Risk of multiple birth associated with in vitro fertilization using donor eggs. *American Journal of Epidemiology*.

[21] Schieve LA, Cohen B, Nannini A (2007). A population-based study of maternal and perinatal outcomes associated with assisted reproductive technology in Massachusetts. *Maternal and Child Health Journal*.

[22] Chavarro JE, Ehrlich S, Colaci DS (2012). Body mass index and short-term weight change in relation to treatment outcomes in women undergoing assisted reproduction. *Fertility and Sterility*.

